# Ontogeny of Myeloid Cells

**DOI:** 10.3389/fimmu.2014.00423

**Published:** 2014-09-03

**Authors:** Ismé De Kleer, Fabienne Willems, Bart Lambrecht, Stanislas Goriely

**Affiliations:** ^1^VIB Inflammation Research Center, University of Ghent, Ghent, Belgium; ^2^Department of Respiratory Medicine, University Hospital Ghent, Ghent, Belgium; ^3^Department of Pulmonary Medicine, Erasmus MC, Rotterdam, Netherlands; ^4^Institute for Medical Immunology (IMI), Université Libre de Bruxelles, Charleroi, Belgium

**Keywords:** neonate, fetus, dendritic cell, monocyte, macrophage, TLR, cytokine, microbiota

## Abstract

Granulocytes, monocytes, macrophages, and dendritic cells (DCs) represent a subgroup of leukocytes, collectively called myeloid cells. During the embryonic development of mammalians, myelopoiesis occurs in a stepwise fashion that begins in the yolk sac and ends up in the bone marrow (BM). During this process, these early monocyte progenitors colonize various organs such as the brain, liver, skin, and lungs and differentiate into resident macrophages that will self-maintain throughout life. DCs are constantly replenished from BM precursors but can also arise from monocytes in inflammatory conditions. In this review, we summarize the different types of myeloid cells and discuss new insights into their early origin and development in mice and humans from fetal to adult life. We specifically focus on the function of monocytes, macrophages, and DCs at these different developmental stages and on the intrinsic and environmental influences that may drive these adaptations.

## Introduction

Granulocytes, monocytes, macrophages, and dendritic cells (DCs) represent a subgroup of leukocytes, collectively called myeloid cells. They circulate through the blood and lymphatic system and are rapidly recruited to sites of tissue damage and infection via various chemokine receptors. Within the tissues they are activated for phagocytosis as well as secretion of inflammatory cytokines, thereby playing major roles in protective immunity. Myeloid cells can also be found in tissues under steady-state conditions, where they control development, homeostasis, and tissue repair.

In this review, we first describe the different types of myeloid cells and their origins in the course of embryogenesis. We then summarize what is known about their functional status in early life and discuss the possible factors that influence their development.

## Origin of Myeloid Cells

### Embryonic hematopoiesis

**Figure 1:**
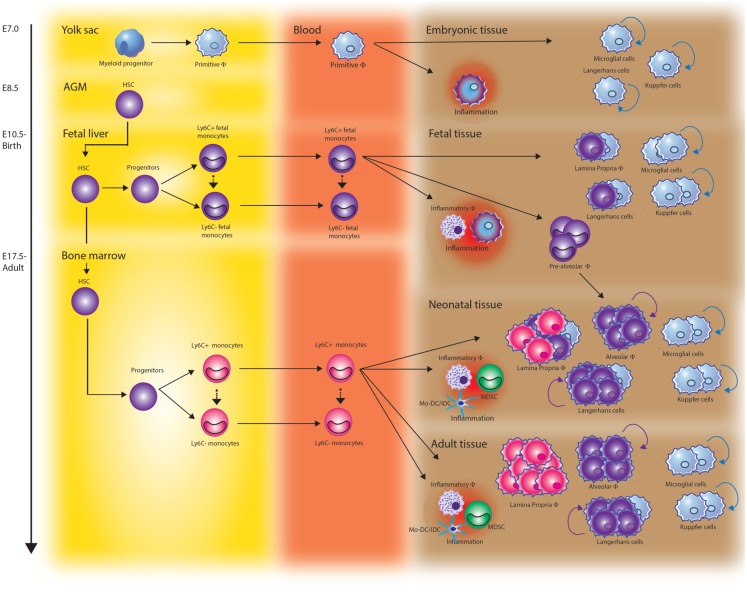
**The origin of monocytes and macrophages throughout development**. Tissue resident macrophages arise at different stages of development and derive from at least three different sources. During early embryonic development, yolk sac-derived myeloid progenitors give rise to microglia in the brain, Kupffer cells in the liver, and Langerhans cells in the skin. Once fetal liver hematopoiesis has started (E10.5 in mice), fetal liver-derived monocytes differentiate into tissue macrophages and contribute to the Langerhans cell pool in the skin and lamina propria macrophages in the gut. They also seed the lung just before birth. After birth, these cells rapidly differentiate into long-lived alveolar macrophages (AMF) via a “pre-AMF,” intermediate differentiation stage. Fetal monocyte-derived Langerhans cells show vigorous proliferation after birth while lamina propria macrophages are continuously renewed via differentiation of bone marrow-derived monocytes. In addition to these resident macrophage populations, Ly6C^high^ monocytes can be recruited to sites of infection or injury and differentiate into inflammatory macrophages, monocyte-derived dendritic cells (Mo-DCs), or myeloid-derived suppressor cells (MDSCs).

Myelopoiesis occurs in mammalians through a stepwise process that begins in the yolk sac (YS) by week 3–4 of gestation in human and on embryonic day 8 (E7) in mice (Figure 1). At this time, long before the generation of definitive hematopoietic stem cells (HSCs), myeloid progenitors develop from the primitive ectoderm of the YS and give rise to embryonic macrophages. This primitive system is followed by definitive hematopoiesis mediated by self-renewing HSCs as the ultimate precursor of the adult hematopoietic hierarchy.

The first HSCs emerging in the embryo derive from the ventral wall of the aorta in the aorta–gonad–mesonephros (AGM) region. At this stage, HSCs can also be found in vitelline/umbilical arteries, the placenta, and YS. Around week 5 of gestation, YS and AGM derived HSCs seed the fetal liver, which is eventually the main fetal hematopoietic site. In the fetal liver, the HSCs expand, mature and, for the first time, give origin to mature erythroid, lymphoid, and myeloid cells (1, 2).

Extra-embryonic hematopoiesis ceases around week 10–12 of gestation in human and E12 in mice. The liver remains the predominant hematopoietic site through 20–24 weeks of gestation in human and until birth in mice. From the fetal liver, HSCs colonize the fetal thymus and spleen, niches that support the expansion of HSCs but do not support *de novo* generation of HSCs. Finally, during the second trimester in human and just before birth in mice the bone marrow (BM) gets colonized, resulting in the production of a small pool of HSCs that are responsible for the maintenance of hematopoiesis throughout adult life (3).

### Monocytes and their early development

Monocytes appear in the fetal circulation as soon as self-renewing HSCs have seeded the fetal liver. HSCs develop through various multipotent progenitor stages into monocyte/macrophage and dendritic cell progenitors (MDP). MDP have lost the ability to generate granulocytes and either give rise to “common monocyte progenitors” (cMoPs) restricted to monocytes and their descendants or commit toward a common DC precursor (CDP) (4, 5). The transcription factor PU.1 plays a prominent role in monopoiesis at various stages of this commitment. High expression of PU.1 activates myeloid-specific factors such as interferon regulatory factor-8 (IRF8), kruppel-like factor 4 (KLF-4), and Erg1 and at the same time antagonizes key regulators of other developmental pathways, such as GATA-1, GATA-2, and C/EBPα. PU.1 is also critical for the expression of the growth factor receptor CD115 (M-CSFR) in MDP as well as during the later stages of differentiation. CD115 and its ligands M-CSF and IL-34 are key regulators of survival, proliferation, and differentiation and indispensable for monocyte development.

Monocytes have long been considered as a developmental intermediate between BM precursors and tissue macrophages. However, renewed interest in recent years has revealed that monocytes carry out specific effector functions during inflammation (6). Monocytes can be divided into two primary subsets based on phenotype and function. The CD14^++^CD16^−^ classical human monocytes or intermediate CD14^++^CD16^+^ human monocytes correspond to mouse GR1^+^/Ly6C^high^ inflammatory monocytes and are CCR2^+^Cx3CR1^low^. The non-classical human CD14^dim^CD16^+^ correspond to the GR1^−^/Ly6C^low^ mouse monocytes and are CCR2^−^ and express large amounts of Cx3CR1. GR1^+^/Ly6C^high^ monocytes and their human CD14^++^CD16^−^ or CD14^++^CD16^+^ counterparts are rapidly recruited to sites of infection or injury and have the potential to differentiate into either inflammatory macrophages or monocyte-derived DC (Mo-DC). They efficiently produce inflammatory mediators such as tumor necrosis factor (TNF)α, nitric oxide, and reactive oxygen species. In mice, these cells have shown to be critical for the control of a number of pathogens, including *Listeria* (7), *Mycobacterium* (8, 9), *Cryptococcus* (10), *Toxoplasma* (11), and herpes simplex virus (HSV) (12). Human CD14^dim^CD16^+^ non-classical monocytes and their mouse Ly6C^low^ equivalents patrol blood vessels and mediate early responses against insult (13–16). These cells have also been shown to promote wound healing and angiogenesis in models of atherosclerosis and cardiac infarction.

Although real evidence is still lacking, an accumulating number of studies indicate that monocytes leave the BM as GR1^+^/Ly6C^high^ monocytes and develop via an intermediate stage into GR1^−^/Ly6C^low^ monocytes. However, most of this evidence stems from adult mouse models and differential pathways via distinct precursors during fetal liver hematopoiesis are not excluded. Indeed, a recent study on the human monocytic equivalents suggested that monocytic cells derived from human embryonic stem cells (hESCs) and fetal liver can differentiate from a CD14^−^CD16^−^ precursor to form CD14^++^CD16^+^ cells without producing the CD14^++^CD16^−^ cell population that predominates in adult peripheral blood (17). In comparison to adult blood monocytes, the embryonic CD14^++^CD16^+^ monocytes secreted high amounts of proteins acting on tissue remodeling and angiogenesis and most of them expressed the angiopoietin-1 receptor (Tie2). This suggests that embryonic and fetal monocytic cells may follow a differentiation pathway different to that of adult cells and leading to specific functions. It remains to determine whether these differences reflect the plasticity of a unique cell population generated by the fetal liver environment or reflect the presence of distinct precursors.

### The origin of macrophages: Yolk sac progenitor or monocyte?

Macrophages are resident phagocytic cells in lymphoid and non-lymphoid tissues with highly diverse roles in the maintenance of an organism’s biological integrity ranging from development, homeostasis, to repair, and immune responses to pathogens. Macrophages exert these functions through clearance of cell debris, production of growth factors, highly efficient phagocytosis, and the production of inflammatory cytokines. Being equipped with a broad range of pathogen-recognition receptors they can act as sentinels and instantly respond to changes in physiology as well as challenges from outside.

Very recently, the concept of monocytes being precursors of tissue resident macrophages has been challenged and the early origin of tissue macrophages reassessed. Using fate-mapping analysis, it was shown that embryonic macrophages around E8.25, when the embryonic heart starts beating, migrate via the nascent circulation to the central nervous system. Here, they form between E8.5 and E9.5 a stable macrophage compartment, the microglia that maintain them without further input from adult hematopoiesis (18–20). YS macrophages also contribute to other adult tissue macrophage populations including liver Kupffer cells (20). The prototypical macrophages of the skin, epidermal Langerhans cells were shown to have a dual origin involving both YS progenitors and fetal liver-derived monocytes (21, 22).

We recently showed that alveolar macrophages (AMF) completely derive from fetal liver-derived monocytes. Although embryonic macrophages colonize the fetal lung around E12.5, we found them quickly outnumbered by high numbers of GR1^+^/Ly6C^high^ fetal liver-derived monocytes entering the lung from E16.5 onward. The fetal liver monocytes differentiate into AMF just after birth and enter the alveolar spaces by 3 days post-natal where they adopt a stable phenotype in response to instructive cytokines and then self-maintain throughout life (23). In contrast to the highly stable macrophage populations in brain and liver and more or less in skin and lungs, the macrophages residing in the intestinal lamina propria are continuously renewed from fetal liver and BM derived GR1^+^/Ly6C^high^ monocytes (24).

Thus, tissue resident macrophages arise at different stages of development and from at least three different sources, i.e., YS macrophages and monocytes derived from fetal liver or BM. In addition to these resident macrophage populations that arise under steady-state conditions and at specific time points, CD14^++^CD16^−^ classical monocytes or their GR1^+^/Ly6C^high^ counterparts in mice can recruit to sites of infection or injury and differentiate into macrophages.

### Trophic function of embryonic macrophages

Once the liver becomes a major source of myeloid cells, it becomes difficult to distinguish macrophages of YS and liver origin in the absence of definitive markers. Therefore, most studies to the function of embryonic macrophages have not made distinction between YS derived embryonic macrophages and macrophages that differentiated from fetal liver-derived monocytes. Regardless of their origin, as in adult mice, embryonic macrophages play a key role in innate responses to pathogens and constitute the primary host defense in the mouse embryo. In addition, several lines of evidence suggest important trophic roles for embryonic macrophages (25, 26). During early development they are most numerous in areas of active tissue remodeling such as the dorsal midline, the developing retina, and interdigital zone in the developing footpad. Programed cell death is an integral part of embryonic development and the macrophages in these regions are actively involved in phagocytosis of dying cells. Embryonic macrophages are also critically involved in allowing primitive erythropoiesis. They are the major source of the red cell growth factor erythropoietin (27) and ingest the nuclei expelled by maturing erythrocytes (28).

Besides erythropoietin, embryonic macrophages have shown to secrete a wide range of other mediators important for regulation of cell function. For example, embryonic macrophages seem to contribute to vascularization of embryonic tissues by secreting appropriate cytokines, a proposal supported by their close association with the developing vasculature. Furthermore, studies of the optic nerve suggest that macrophages produce factors that are necessary for axon growth and guidance. Embryonic macrophages also have been implicated in depositing components of basement membrane such as proteoglycans, laminin, tiggrin, type IV collagen, and glutactin (29).

Collectively, these data suggest that embryonic macrophages as well as fetal liver-derived monocytes have a major function in tissue growth and remodeling. This conclusion is underscored further by studies showing that the gene signature of mouse embryonic macrophages is shared with the so-called tumor-associated macrophages (TAM) that have tumor remodeling and immunosuppressive functions (30).

### Conventional DCs

Dendritic cells are specialized antigen-processing and -presenting cells. By taking up antigen and bringing it to tissue draining lymph-nodes DCs have major functions in the initiation and regulation of adaptive immune responses and are central to the development of immunological memory and tolerance. In recent years, researchers have defined distinct DC subsets differing in surface marker expression and development (31, 32). In the adult situation all lymphoid tissue, including spleen, LN, and BM, as well as most non-lymphoid tissue contain two families of conventional DCs in the mouse characterized by either CD8α and CD103 or CD11b integrin expression and equivalents of human CD141/BDCA3^+^ cDC and CD1c/BDCA1^+^ cDC. CD8α^+^ cDCs and CD103^+^ cDCs are functionally specialized in cross-presentation of exogenous viral antigen to CD8^+^ T cells (33, 34). CD8α^+^ cDCs also present glycolipid Ags in CD1d context and can activate and polarize iNKT toward the production of T helper 1 (Th1) or Th2 cytokines (35). Conversely, CD11b^+^ DCs seem specialized in presenting soluble antigen to CD4 T cells (36) and produce large amounts of pro-inflammatory chemokines such as CCL3, CCL4, and CCL5 (37–39). This specialization of CD11b^+^ cDCs was recently attributed to their expression of the transcription factor IRF4.

In mice, DCs arise from CDPs that expresses the hematopoietic cytokine receptor Flt3 and gives rise to Plasmacytoid DC (pDC) or pre-conventional DCs (pre-cDCs) (4, 40). The transcription factors PU.1, Gfi1 (41), and Cbfb (42) control the development of the common DC lineage. Pre-cDCs can be found in the blood and develop further in the tissue into the two subsets of cDCs. It is unknown whether committed DC progenitors exist in humans. So far, no equivalents of mouse MDP, CDP, or pre-DC have been identified. Development of CD8α^+^ cDCs and CD103^+^ cDCs from pre-DCs is orchestrated by the same transcription factors: inhibitor of DNA binding 2 (Id2), IRF8, basic leucine zipper ATF-like 3 transcription factor (BATF3), and the nuclear factor interleukin 3 regulated (NFIL3). CD11b^+^ cDC development is controlled by transcription factors including RelB (43), NOTCH2 (44), RBP-J (45), IRF2 (46), and IRF4 (47).

Conventional DCs generally display a short half-life of approximately 3–6 days and in adults are constantly replenished from BM precursors in a strictly Flt3L-dependent manner (48). So far, splenic DC development has not been investigated during fetal life. At birth, DCs represent only 0.2% of murine splenic cells and reach adult levels by 3 weeks of age (49, 50). A similar post-natal development of DCs is described in murine as well as human neonatal lungs (51, 52).

### Non-conventional DCs: Plasmacytoid DCs and monocyte-derived DCs

Plasmacytoid DCs are present in the BM and all peripheral organs. They are relatively long-lived and display a characteristic surface phenotype and morphology, including a highly developed secretory compartment (53). pDCs are specialized to respond to viral infection with a massive production of type I interferons (IFNs). They can also act as antigen-presenting cells and control T cell responses (54–56). Suppression of Id2, the transcription factor critical for cDC development, by E2-2 (also known as TCF4) leads to pDC development (57, 58).

As a consequence of inflammation or infection, lymphoid and non-lymphoid organs can also harbor DCs that originate from monocyte infiltrates and have been termed “monocyte-derived DCs” (MoDCs) or “inflammatory DCs” (iDCs) (59–61). For a long-time MoDCs have been phenotypically difficult to discern from cDCs because they share similar expression patterns of MHC-II, CD11b, and CD11c. However, recent studies have identified CD64, the Fc-gamma receptor 1 (FcγRI) as a Mo-DC marker in the mouse (62, 63) and indicated that Mo-DCs, through their rapid and numerous recruitment and high production of chemokines and inflammatory cytokines play an important role in the initiation of inflammation (62, 64–66).

### Granulocyte development

Granulocytes are at all ages the most abundant type of myeloid cells in the blood stream and can be further subdivided into neutrophils, eosinophils, and basophils. All granulocytes derive from the granulocyte/monocyte progenitor (GMP), through further differentiation into the eosinophil lineage-committed progenitor (EoP), and the basophil/mast cell progenitor (BMCP), which in turn gives rise to the mast cell progenitor (MCP) and the basophil progenitor (BaP). With regard to neutrophils a committed progenitor is not yet described. Granulopoiesis is present in the liver parenchyma of human fetuses as early as 5 weeks gestation and is dependent on the transcription factors C/EBPα, PU.1, and GATA-2.

In adults, neutrophils are the most frequent granulocytes. They are constantly generated in a high number in the BM and circulate with the blood stream until activated by signals that are provoked by resident macrophages at the site of infection or injury. Once in the tissue neutrophils combat microorganism via phagocytosis, the release of microbicidal proteins and by neutrophil extracellular trap formation. Until the third trimester fetal blood contains few neutrophils. Although mature neutrophils are scarce, progenitor cells with the capacity to generate neutrophil clones are abundant in fetal blood. The production of GM-CSF and G-CSF, the cytokines that drive differentiation of precursors into granulocytes and promote the survival of mature neutrophils is also low in fetal blood. However, G-CSF shows a steep increase just before birth, most likely contributing to the quick rise in neutrophils seen in the same period. This kinetics also fits the finding that G-CSF receptors on the surface of neutrophils of newborn infants are equal in number and affinity to those on adult neutrophils (67).

Eosinophils are resident in various organs such as the gastrointestinal tract and BM and contribute to tissue and immune homeostasis. Only a minor part of the eosinophils circulates in the peripheral blood and is recruited mainly upon TH2 responses into sites of inflammation. Within the tissues they produce several cytokines and lipid mediators and release toxic granule proteins. Eosinophils are associated with immune responses directed against parasites or allergens and contribute to immune pathology and parasite clearance (68). Eosinophilopoiesis is observed in the fetal liver as early as 5 weeks after gestation. Fetal liver eosinophils still have self-renewing capacity although they already lost the classical stem cell markers (c-Kit, CD34, and Sca-1). The cytokines IL-3, IL-5, and GM-CSF are especially important for eosinophil expansion. Of these three cytokines, IL-5 is the most specific to the eosinophil lineage and is responsible for selective differentiation of eosinophils. Besides neutrophils cord blood contains more mature eosinophils as well as more progenitor cells than adult peripheral blood; thus, neonates seem to have a high capacity to produce high eosinophil counts (69, 70). It is also widely known that premature infants develop eosinophilia during the first weeks of post-natal life (71, 72). The physiological role of this phenomenon is not yet understood.

The least common granulocytes in the circulation are basophils. Basophils play a central role in inflammatory and immediate allergic reactions. They are able to release potent inflammatory mediators, such as histamine, proteases, chemotactic factors, cytokines, and metabolites of arachidonic acid that act on the vasculature, smooth muscle, connective tissue, mucous glands, and inflammatory cells.

## Functional Status of Monocytes, Inflammatory DC, and Macrophages in Early Life

As discussed in the previous sections, the first days/weeks of post-natal life in mice is an important transition period for the ontogeny of monocyte/macrophage system this reflects the sources of progenitors (different waves from the YS, the liver, and the BM), the local production of growth factors and the influence of the first encounter with microorganisms from the microbiota. It is therefore not surprising that the functional capacity of macrophages gradually changes during this period. For example, in young rats, AMF display a lower capacity for phagocytosis and cytokine production (73). Epidermal Langerin^+^ cells proliferate intensely during the first week of life (21). These cells display reduced capacity to activate T cells and low expression of costimulatory molecules (74).

Due to the longer intra-uterine life, post-natal development of myeloid cell populations in humans might be less important in term infants than in rodents. Recently, distinct functional characteristics were identified between human adult and fetal monocytes by comparing the transcriptional and signaling programs of classical monocytes from fetal (18–22 gestational weeks) and adult BM (75). Interestingly, fetal monocytes phosphorylate canonical and non-canonical STATs and respond more strongly to IFNγ, IL-6, and IL-4 than adult monocytes. Upon stimulation with IFNγ, fetal monocytes fail to upregulate costimulatory and antigen presentation genes but instead upregulate many genes, which mediate innate pathogen responses (75).

As far as neonatal human macrophages are concerned, they exhibit decreased responsiveness to IFNγ, which is associated to a marked alteration in Stat1 phosphorylation (76). A decreased phagocytic activity of *E. coli* was detected in neonatal macrophages compared to adult cells, this alteration being even more pronounced in fetuses before 30 weeks of gestation (77).

Most of our understanding on human myeloid cell function in early life arises from *in vitro* data obtained with cord blood cells. In terms of cytokine production elicited by TLR ligation, human cord blood mononuclear cells have been found to produce less IL-1α, IL-1β, TNFα, IL-18, and IL-12p70 but equal or greater amounts of IL-6 or IL-10 compared with adult cells. Single cell flow cytometry analysis of cytokine production revealed that cord blood monocytes produced less TNFα but as much or even more IL-12/23p40 and IL-6 in response to TLR2, TLR-4, and TLR7/8 ligands in comparison to adult monocytes (78). Furthermore, TLR-mediated production of innate immune effector molecules such as oxygen radicals is profoundly attenuated in early life (77). These findings show that TLR-mediated responses of human neonatal monocytes are not globally impaired or altered but follow distinct rules from that of control cells from adults. Although previous studies have indicated reduced expression of TLR-4 on cord blood monocytes compared to its expression in adults, other studies have not. A reduced expression of TLR-4 was recently detected on cord blood monocytes as a consequence of a reduced frequency of intermediate CD14^++^CD16^+^ monocytes in cord blood compared to the frequency in adult blood of such subset characterized by high TLR-4 expression (79). In comparison to classical CD14^++^CD16^−^ and non-classical CD14^dim^CD16^+^ monocytes, intermediate CD14^++^CD16^+^ monocytes represent the main producers of TNFα in response to microbial products. Their reduced frequency in cord blood could therefore contribute to LPS hyporesponsiveness in newborns. At the signaling level, a decreased expression of MYD88, as well as a reduced NF-κB-dependent transcriptional activation was observed in neonatal monocytes as compared to adult counterparts (80).

As mentioned earlier, monocytes can differentiate *in vivo* into iDCs as a consequence of inflammation or infection and *in vitro* in presence of GM-CSF and IL-4 or by migrating through the endothelium (81, 82). Cord blood MoDCs were found to produce very low levels of IL-12p70 in response to LPS, poly I:C, *Bordetella pertussis* toxin or CD40 ligation (83–85). This limited production of IL-12p70 is due to a specific decrease in the transcription of the IL-12p35 subunit, while the IL-12p40 subunit transcription is preserved. Moreover, expression of IFNβ and IFN-inducible genes such as CXCL9, CXCL10, and CXCL11 are selectively reduced in LPS-stimulated cord blood cells in comparison to adult cells (86). A low IFNβ production was associated with a decreased expression of interferon regulatory factor (IRF)3-dependent genes but not of NF-κB-dependent genes, indicating that TRIF-dependent signaling events are preferentially affected in neonatal cells despite comparable levels of TLR-4 expression in adult and neonatal MoDCs. While proximal signals leading to IRF3 activation are preserved, its interaction with CBP are altered in neonatal DCs, leading to impaired DNA binding capacity (86). In addition to its critical role in TLR-4-mediated IFNβ synthesis, IRF3 is also directly involved in IL-12p35 gene expression (87). Interestingly, reduced IL-12p70 synthesis in neonatal MoDCs was associated with impaired chromatin remodeling in the IL-12p35 promoter region (88). It is therefore possible that limited expression of IL-12p35 subunit in LPS-stimulated neonatal MoDCs could be due to altered recruitment of the IRF3/CBP complex to the IL-12p35 promoter.

The observation that the NF-κB-dependent pathway in TLR-4 signaling is intact in neonatal MoDCs is consistent with their ability to produce pro-inflammatory cytokines upon LPS stimulation such as TNF-α, IL-6, IL-8, and IL-23, which all depend on NF-κB.

Altogether, these findings highlight the differential signaling pathways underlying distinct and unique patterns of inflammatory responses in neonatal and adult monocytes or MoDCs. How these differences may participate to the high burden of infectious diseases in early life is still a matter of debate (89). The high susceptibility to specific pathogens in newborns and infants results from many different factors, including immunological, anatomical, genetic, and environmental factors. It is tempting to speculate that the decreased capacity of circulating monocytes to produce inflammatory cytokines such as TNF-α or IL-1β predisposes premature (and term) newborns to widespread bacterial sepsis. Decreased capacity to produce IL-12p70 is long-lasting and correlates with the susceptibility of young children to disseminated infections with pathogens that require efficient Th1-type responses, such as *Mycobacterium tuberculosis, Salmonella*, or *Burkholderia pseudomallei* (89). Furthermore, in comparison to adult controls, antigen-specific IFN-γ production was found to be decreased upon vaccination with oral polio or hepatitis B vaccines at birth (90, 91). However, BCG vaccination at birth elicits higher IFNγ and IL-17 production than when the immunization is achieved at 4 months of age (92). These observations re-enforce the notion that the immune system in early life can be efficiently activated under specific conditions.

## Functional Status of Conventional and Plasmacytoid Dendritic Cells in Early Life

As discussed in the previous sections, development of most myeloid cell populations, including DCs is well advanced in human term infants. The situation is different in newborn mice where the splenic composition of DC subsets varies significantly from the adult. Indeed, at birth, pDC and CD4^−^CD8α^−^cDC are found in the spleen whereas CD8α^+^ and CD4^+^ cDC are not present (49, 50). The CD4^+^ cDC subset predominates by the age of 3 weeks whereas a significant number of CD8α^+^ cDC accumulate in the spleen by day 6 after birth. This particular cDC subpopulation is endowed with a high capacity to produce IL-12. Hence, preferential polarization into Th2 cells observed upon immunization in the first week of life was attributed to the delayed maturation of CD8α^+^ cDCs in the spleen (93). IL-12 levels produced by *ex vivo* isolated splenic cDC from 7-day-old mice were found to be either similar or reduced in comparison to their adult counterparts (49, 50, 94). The differences could be due to the mode of stimulation as CpG was used either alone or in combination with cytokines. Whatever the reason for this difference, such a delayed maturation of IL-12p70 production also occurs in humans. Indeed, longitudinal studies using whole blood or peripheral mononuclear cell cultures revealed age-dependent changes in TLR-induced cytokines in cDC in infancy. As observed in MoDCs, cDCs synthesize very low levels of IL-12p70 but much higher levels of IL-23 in comparison to adult cells. After TLR stimulation, production of IL-12p70 is still below adult levels in 12 years old children whereas synthesis of IL-23, IL-6, and IL-10 dominates in term infants (78, 95, 96). Interestingly, production of IL-23, IL-6, and IL-10 declines over the first few years of life, while secretion of pro-inflammatory cytokines TNFα and IL-1β gradually increases with age (97, 98). These findings indicate that cDCs have a reduced ability to produce Th1-supporting cytokines, which corresponds to increased risk of infection with intracellular pathogens such as *Listeria monocytogenes, M. tuberculosis*, and HSV in early life. Conversely, at birth, human cDCs have an enhanced capacity to promote Th17-type immune responses involved in the defense against extracellular pathogens (99). Interestingly, cord blood naïve CD4^+^ T cells from preterm and term infants have the potential to develop into Th17 effector cells upon *in vitro* stimulation under Th17 polarizing conditions (99). Th17 responses can be detected in the peripheral blood and the airways of respiratory syncytial virus (RSV)-infected 1-month-old infants suggesting that the capacity for Th17 development may be acquired quickly after birth (100).

Plasmacytoid DCs have the unique property to sense a variety of viruses by pattern recognition receptors and to secrete very rapidly 10–100 times more IFN (IFN)- αβ than other immune cells. Despite comparable levels of TLR9 and TLR7 expression in adult and neonatal human pDCs, cord blood pDCs exhibit a strong limitation in their capacity to produce IFNαβ in response to TLR9 as well as TLR7 ligation, HCMV, or HSV-1 exposure (101). This decreased production is associated with a reduced nuclear translocation of IRF7 (102). In contrast, a recent report indicates that purified cord blood pDCs are responsive to CpG and a variety of viruses (103). Despite such discrepancy, impaired type I IFNs production was demonstrated both in whole cord blood cultures after stimulation with CpG or R848 and *ex vivo* in pDC from cord blood mononuclear cells infected with RSV, showing that human pDCs are clearly less functional in early life (104). Similarly, in mouse, neonatal pDCs exhibit dampened IFNα and IRF7 translocation during lymphocytic choriomeningitis virus (LCMV) infection, which was correlated with downregulation of E2.2, a master transcription factor regulating pDC development and function (105). The fact that neonatal murine pDCs were found to display an adult-like response capacity when assessed *in vitro* indicates that the decreased functionality of murine pDCs is not cell-autonomous but reflects the influence of their local environment (see below). Altogether, these observations suggest that early life pDCs responses are tightly regulated *in vivo*, which may be beneficial to avoid potentially harmful inflammatory or autoimmune reactions and resulting in increased vulnerability to viral pathogens such as influenza, RSV, HSV-1, or cytomegalovirus (CMV).

## Potential Mechanisms Involved in the Post-Natal Acquisition of Adult-Like Function by Myeloid Cells

**Figure 2:**
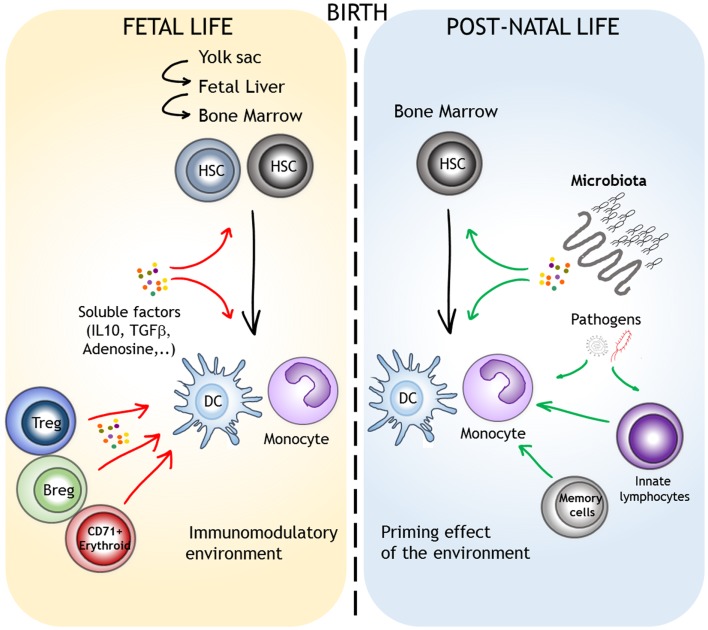
**Factors that can account for the different functional properties of monocytes/DCs in the course of their ontogeny**. Monocytes and DCs are highly plastic, even in “steady-state” conditions. Lineage specification and function are influences by the origin of the progenitors and by environmental factors. We depict the role of the materno-fetal immunomodulatory environment (red arrows) and the direct or indirect priming effects of microbial-derived signals that are encountered after birth (green arrows).

It is generally believed that there is an inherent immaturity in the myeloid compartment in newborns and young infants that contributes to their susceptibility to infections and impairs their responses to vaccination. As discussed above, this might be the case in rodents where post-natal period is still important for the colonization of lymphoid and non-lymphoid organs by liver and BM myeloid progenitors. In humans, monocytic cells derived from embryonic and fetal liver progenitors preferentially express M2-type signature genes (17). In comparison to their adult counterparts, BM monocytes from mid-gestational age also display distinct responsiveness to cytokine stimulation. These results imply that the functional differences observed in early life could result from a distinct origin of precursors. However, it is not clear when the transition between fetal (liver) versus “adult-like” (BM) myeloid progenitors occurs in human ontogeny. Colonization of BM by hematopoietic progenitors starts around 15–16 weeks of gestation (106). High levels of circulating HSCs are observed before 32 weeks of gestation, probably reflecting the active transfer of hematopoiesis from liver to BM (107). Hence, in term newborns, BM probably represents the major source of myeloid progenitors. Yet, at birth, the function of these different subpopulations appears to be qualitatively and quantitatively different from that of adults. The classical model of hematopoiesis is based on a hierarchy of progenitors that progressively lose their developmental potential (108). It is not evident to apply this model to monocyte/macrophage/DC differentiation as data from patients with mutations in GATA-2 or IRF8 reveal the intricate relationship between the various “committed” progenitors that allows the convergence of different paths to the same cell types (109). These concepts allow a high degree of functional plasticity in response to micro-environmental cues throughout the differentiation process. Hence, multiple factors can account for the distinct functional properties of myeloid cells in early life (Figure 2). The materno-fetal environment is very specific. High local or systemic levels of immunomodulatory factors such as IL-10, TGFβ, or adenosine can directly affect the function of myeloid cells during fetal life or early post-natal life (110). Several populations with immunosuppressive functions were suggested to participate to materno-fetal tolerance. These include regulatory T cells (Tregs), B cells (Bregs), CD71^+^ erythroid cells, or myeloid-derived suppressor cells (MDSCs) (111–114). The most dramatic event that occurs at birth is certainly the initial colonization of the gastrointestinal tract by the microbiota. Strikingly, macrophages isolated from mice treated with antibiotics show reduced expression of IFN-responsive genes, suggesting that signals derived from commensal bacteria influence systemic innate antiviral responses (115). Furthermore, this might implicate tunable chromatin level changes as DCs from germ-free or antibiotic-treated mice show reduced H3K4me3 deposits at specific inflammatory genes (116). Despite normal NF-κB or IRF3 activation upon TLR stimulation, direct recruitment to promoter regions was reduced, an observation that parallels previous findings in cord blood monocyte-derived DCs. Tonic stimulation by commensals might therefore enable rapid induction of specific defense genes upon infection, a hypothesis that goes along well with the concept of “trained immunity” (117). Such mechanism could account for blunted type I IFN and IL-12 production by cord blood DCs and alterations of STAT signaling pathways in fetal monocytes and macrophages. The way microbial-derived signals influence the function of immune cells even at distant sites is still poorly understood. Soluble factors such as metabolites can clearly play a determinant role in this process. Constant diffusion of low-level microbial products such a TLR or NOD ligands from the gastrointestinal tract to the bloodstream could also drive this bystander control of peripheral responses (118). Interestingly, such mechanisms would then allow the maternal flora to influence the function of fetal innate immune cells during gestation. This systemic effect could, however, be indirect, for example, by triggering the local activation of innate lymphoid cells.

Several reports indicate that the first year of life represents an important period for the acquisition of “adult-like” responsiveness to TLR ligands by circulating monocytes/DCs. Major changes in the composition of the gut and respiratory tract microbiota occur during this period. The development of the microbiota is influenced by multiple factors such as the mode of delivery, the feeding regime, or the use of antibiotics (119). Along this line, decreased diversity of the bacteroidetes phylum in infants born by cesarean section is associated with lower seric levels of CXCL10 and CXCL11, two IFN-dependent chemokines (120). Thus, defined members of the microbiota are likely to influence the trajectory of myeloid cell function in the course of childhood.

In mice, splenic DCs are entirely replaced by circulating progenitors within 2 weeks (121). One would therefore expect the “adjuvant” effect of commensals on myeloid function to be transient and reversible. However, other myeloid subpopulations such as tissue macrophages or Langerhans cells are long-lived and arise from local progenitors. This implies that exposure to other microbial-derived signals, in the context of inter-current infections or vaccination could continuously shape the function of myeloid cells throughout life. Once again, these effects could directly target myeloid cells or influence their function through the activation and expansion of innate lymphocytes such as NK or γδ T cells (122–124). The capacity to produce IL-12 slowly increases with age, suggesting that a maturation process occurs throughout life, a phenomenon that parallels the acquisition of memory T and B cells.

In conclusion, our knowledge of the origin and development of myeloid cells during embryogenesis and early life has greatly expanded in recent years. The functional capacity of myeloid cells to respond to pathogens is influenced by the origin of their precursors and also by non-cell-autonomous factors such as signals derived from the commensal microbiota. Defining the molecular and cellular mechanisms underlying the determinants of myeloid cell functions in early life will allow a better understanding of the susceptibility of young infants to infections and other clinically relevant implications such as the development of allergies.

## Conflict of Interest Statement

The authors declare that the research was conducted in the absence of any commercial or financial relationships that could be construed as a potential conflict of interest.
